# Challenges Reported by Certified Nursing Assistants During COVID-19: A Qualitative Study

**DOI:** 10.1093/geroni/igab046.957

**Published:** 2021-12-17

**Authors:** Joann Reinhardt, Emily Franzosa, Wingyun Mak, Orah Burack

**Affiliations:** 1 The New Jewish Home, New York, New York, United States; 2 Icahn School of Medicine at Mount Sinai, Icahn School of Medicine at Mount Sinai, New York, United States

## Abstract

Certified nursing assistants (CNAs) who care for vulnerable nursing home residents faced unprecedented circumstances due to the COVID-19 pandemic. While staff and PPE shortages were ubiquitous and widely known, the focus of this qualitative work was to gain a broader understanding of the numerous challenges they faced. We conducted 10 remote focus groups with CNAs at 5 nursing homes (N=56) in downstate New York. Content analysis was conducted, and emerging themes identified. Results showed a myriad of emotional challenges including helplessness, fear and anxiety. Operational challenges focused on lack of COVID testing capacity, information, and consistent guidance and support, in addition to staff and equipment. Individual challenges included personal experience of COVID illness and that of colleagues, and balancing high concurrent demands of work and family. These results are discussed in the context of developing recommendations to promote future safety, skill refinement and enhanced resilience in the workforce moving forward.

